# Development of the covalent antibody-DNA conjugates technology for detection of IgE and IgM antibodies by immuno-PCR

**DOI:** 10.1371/journal.pone.0209860

**Published:** 2019-01-04

**Authors:** Artem V. Maerle, Maria A. Simonova, Victor D. Pivovarov, Daria V. Voronina, Polina E. Drobyazina, Dmitriy Yu. Trofimov, Leonid P. Alekseev, Sergei K. Zavriev, Dmitriy Yu. Ryazantsev

**Affiliations:** 1 OOO DNA-Technology TS, Moscow, Russia; 2 Shemyakin-Ovchinnikov Institute of Bioorganic Chemistry, Russian Academy of Sciences, Moscow, Russia; 3 Federal state budget institution “National medical research center for obstetrics, gynecology and perinatology named after academician V.I. Kulakov” of the Ministry of healthcare of Russian Federation, Moscow, Russia; 4 NRC Institute of Immunology, Federal Biomedical Agency of Russia, Moscow, Russia; University of Houston, UNITED STATES

## Abstract

Immuno-PCR (iPCR) is one of the methods used for the detection of a wide range of analytes and features the high sensitivity of the polymerase chain reaction (PCR) method. iPCR uses antibodies coupled to DNA, followed by the amplification of the attached DNA using RT-PCR. Two major types of antibody-DNA conjugates are currently used, which are obtained as a result of non-covalent (biotin-streptavidin) or covalent interactions. Using a strain-promoted azide-alkyne cycloaddition (SPAAC), we synthesized covalent DNA-antibody conjugates, optimized the reaction conditions, and developed an efficient protocol for the purification of conjugates, with which all unreacted antibodies and oligonucleotides are separated. Covalent DNA-antibody conjugates were tested with iPCR assays that were previously developed for the detection of IgE and IgM antibodies with the use of the supramolecular complex of 5'- and 3'-biotinylated DNA and streptavidin. The results show that the modification of antibodies with amino groups did not allow us to obtain monolabeled antibodies or antibodies with a strictly defined number of DNA-labels. The degree of labeling determined by the dyes introduced through the azido group reflects the actual labeling degree statistically. If the average labeling degree for azido groups is 1.1, the conjugates contain 25% mono-labeled antibodies, 50% double-labeled antibodies, and 25% unlabeled ones. The specificity of the monoclonal antibody to human IgE (BE5) changed after conjugation with the oligonucleotide. The sensitivity of iPCR in the detection of IgM antibodies produced against the Le^C^ disaccharide using a covalent conjugate was similar to that of a supramolecular complex of 5'- and 3'-biotinylated DNA and streptavidin, but the new procedure is two steps shorter.

## Introduction

Immuno-PCR (iPCR) is a highly sensitive method for the detection of a wide range of analytes ranging from bacterial and viral antigens and antibodies to non-protein drugs and toxins. It combines advantages of both the polymerase chain reaction (PCR) and enzyme immunoassay methods. iPCR is based on the usage of an antibody-DNA conjugate followed by the amplification of the DNA label [1]. DNA-antibody conjugates are currently used for iPCR, immuno-RCA (immunoassay with rolling circle DNA amplification) [2], proximity ligation assay (PLA) [3], and Electrochemical Proximity Assay (ECPA) [4].

The main methods for the preparation of conjugates are based on either biotin-streptavidin interaction or covalent binding. When biotin-streptavidin conjugates are used, biotin is introduced into both the antibody and the DNA label. In the case of iPCR, after applying of detecting antibodies, a solution of streptavidin is added to the wells of a plate, followed by a biotin-containing DNA label. Thus, the antibody-streptavidin-DNA conjugate is formed during analysis. This approach is known as "universal iPCR" and is popular for its simplicity [5]. However, an obvious disadvantage of the method is the three-stage assembly of the conjugate during analysis, which increases the analysis time and the number of washings.

A ready-to-use antibody-DNA conjugate removes this disadvantage. The following methods are used to prepare covalent conjugates for iPCR:

1. Maleimide group + sulfhydryl group (for instance, antibody modification using SMCC (succinimidyl 4-(N-maleimidomethyl) cyclohexane-1-carboxylate) and conjugation with thiol-modified oligonucleotide [6]).
2. Aromatic hydrazine + aromatic aldehyde (for instance, antibody modification using 4-hydrazinonicotinate acetone hydrazone (SANH) and introduction of aldehyde group into oligonucleotide [7]).
3. Oxidation of the antibody followed by reaction with hydrazine group of the modified oligonucleotide [7].
4. Alkyne + azide. Copper(I)-catalyzed alkyne-azide cycloaddition is most widely used in "click" chemistry. However, due to possible denaturation of proteins under the influence of monovalent copper ions [8], copper-free click is used for bioconjugation. Gong et al. describe the antibody modification with a hydrophilic derivative of dibenzocyclooctyne (DBCO-PEG5-NHS) and an azidated oligonucleotide (SPAAC) [9].

The iEDDA reaction (inverse electron demand Diels–Alder reaction) between tetrazine and trans-cyclooctene (TCO) has one of the fastest reaction constants among bioorthogonal reactions and has also been used for conjugation [10]. Antibody was modified with tetrazine using succinimidyl ester (NHS-s-s-PEG_4_-tetrazine with a linker cleavable upon reduction of the disulfide), and the azidated oligonucleotide was modified by trans-cyclooctene via dibenzocyclooctyne. Bertozzi described strain-promoted azide-alkyne cycloaddition (SPAAC) or the so-called copper-free click for the first time in 2004. This method has replaced the canonical reaction catalyzed by copper due to the toxicity and protein denaturation of copper [11]. The introduction of strained cyclooctyne to the reaction with azides improves the reaction (the reaction rate constant is 0.1–1.0 M^-1^ s^-1^), additional reagents are not required [12–16]. However, for the bioconjugation of proteins using SPAAC, it is necessary to preliminarily introduce azide or cyclooctyne residue into the protein molecule of interest.

Kits for the preparation of conjugates for iPCR are commercially available, such as the Solulink Antibody-Oligonucleotide All-in-One Conjugation Kit (based on the aromatic hydrazine + aromatic aldehyde reaction) and the Innova Thunder-Link kit. A number of studies describe the preparation of conjugates and their application in iPCR. However, almost none of them described the technical details of the preparation and purification. In the present study, we synthesized the antibody-oligonucleotide conjugate by a strain-promoted azide-alkyne cycloaddition for use in iPCR. We focused on the optimization of the conditions for the preparation and isolation of conjugates with a certain number of labels.

## Materials and methods

### Sera, allergens, and antibodies

Monoclonal mouse antibodies to human IgM (MA2) and human IgE (BE5) were purchased from Sorbent Ltd, Russian Federation. Svirshchevskaya et al. describe the sera and the preparation of the recombinant allergen Alt a 1, as well as the biotinylation of the BE5 antibodies [17]. The BE5 binding was detected by sheep anti-mouse antibody HRP conjugate (Dako, Sweden).

### iPCR using DNA-streptavidin complex

iPCR was performed using DNA-streptavidin complex in accordance with previous studies for the detection of IgE [18] and the detection of antibodies to Le^C^ [19].

### Oligonucleotides

5’-CgTgCCgCTgTCTCTACCAT-NH_2_-3’ and ODN Ip3-am 5’-AGGCGAACTGTTTTGGTCATAaCCCGCTACTGATTGTTCGCACGGaGTGCTGTGCTTGTGTAAGG-NH_2_-3’) were synthesized using standard solid-phase phosphoramidite chemistry (Synthesizer AB3400 (Applied Biosystems, USA), solid support 3'-Amino-Modifier C7 CPG 1000 (Glenresearch, USA)). PCR primers (AGGCGAACTGTTTTGGTCATA and CCTTACACAAGC ACAGCAC) and the fluorescent probe (5’-BHQ-1 CCCGCTACTGATTGTTCGCACGG FAM-3’) were manufactured by Lumiprobe, Russia.

### Antibodies and oligonucleotide modification

**Fig 1:**
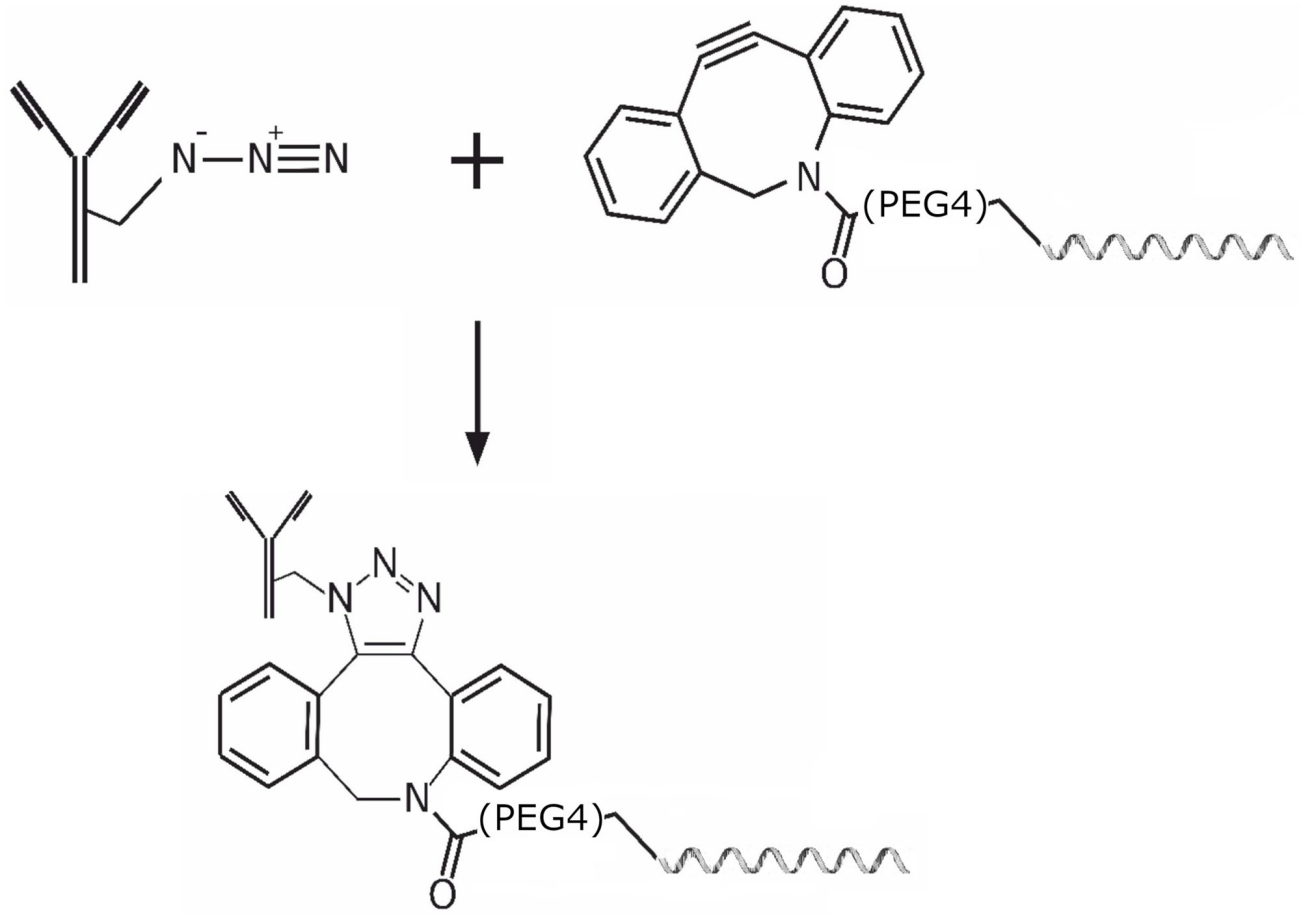
Synthesis of covalent DNA-antibody conjugate.

A strain-promoted azide-alkyne cycloaddition was used to obtain an antibody and single-stranded 60-base oligonucleotide (ODN) conjugates (see Fig 1). Pentynoic acid sulfotetrafluorophenyl ester (STP-N3) (Lumiprobe) was used as a modifying agent for the antibody. The N-hydroxysuccinimide ester of dibenzocyclooctyne with hydrophilic linker (DBCO-PEG_4_-NHS, Jena Bioscience) was used to modify the oligonucleotide with the amino group introduced during synthesis.

### Antibody modification (general scheme)

The antibody was transferred into 0.1 M sodium bicarbonate at pH 8.3 and simultaneously concentrated by washing 3 times with 650 μl of buffer solution on a Spin-X UF 30K MWCO column (Corning). Different excesses of STP-N3 were added to 25 nmol of antibody, and the reaction mixtures were incubated at room temperature for 1 h. The excess reagent was removed using CentriPure MINI PBS Z-50 Spin Columns. The activity of the antibodies after azidation was examined using ELISA. The completeness of the reaction was controlled as follows. An aliquot of modified antibody was mixed with a tenfold molar excess of dibenzocyclooctyne derivative of sulfo-Cy5 (DBCO-Sulfo-Cy5, Jena Bioscience), followed by purification using gel filtration. The labeling level was calculated by analyzing the UV-vis spectra.

### Oligonucleotide modification (general scheme)

Acetone/lithium perchlorate was used to precipitate 100 nmol of ODN Ip3-am, which was then dissolved in 100 μl of buffer containing 0.1 M NaHCO_3_ at pH 8.3 and 80% DMSO. Next, 50 μl of 100 mM DBCO-PEG_4_-NHS (5 μmol) was added, and the reaction mixture was incubated at room temperature for 1 h. The oligonucleotide was precipitated using acetone/lithium perchlorate and purified by gel-filtration on an EMP CentriPure N2 while simultaneously transferring it into a PBS solution. An aliquot of the reaction products was analyzed using RP HPLC (Phenomenex Luna 5u C8, linear gradient of acetonitrile in 50 mM sodium acetate; results are not provided). The reaction products were evaporated using a vacuum centrifuge and dissolved in 50 μl of PBS. The reaction progress was controlled by mixing an aliquot of modified oligonucleotide with a 10–20× molar excess of azido derivative of sulfo-Cy5 (sCy5-N3) (Lumiprobe, Russian Federation), followed by purification using RP HPLC.

### Synthesis and purification of covalent DNA-antibody conjugates (general scheme)

The modified antibody and oligonucleotide were mixed at a ratio of 1:4 by active groups (the determination of the active groups’ content is described below). The reaction mixture was kept at room temperature overnight. The purification was carried out in several stages. To remove the unreacted antibody, the reaction mixture was placed in a RESOURCE Q anion exchange column (GE Healthcare). Chromatography was carried out in 50 mM TrisHCl at pH 7.5 with a linear gradient of NaCl and a flow rate of 1.5 ml/min. The resulting fractions were concentrated and placed in a Superdex 200 10/30 Increase column (GE Healthcare). Chromatography was carried out again in 0.1× PBS buffer at a flow rate of 0.5 ml/min. The fractions were evaporated in a vacuum centrifuge and analyzed by SDS-PAGE in 6% gel (in non-reducing conditions without the addition of mercaptoethanol) and 10% gel (in reducing conditions with the addition of mercaptoethanol), followed by silver staining.

### iPCR analysis with covalent DNA-antibody conjugate

iPCR was performed with direct DNA-antibody conjugate as reported previously [18–20] except for the conjugate addition stage. The IgE analysis was carried out by coating plate wells with the recombinant allergen Alt a 1 in carbonate-bicarbonate buffer at pH 9.6 with a concentration of 2 μg/ml (50 μl per well). The coating was carried out overnight at +4°C. Next, the wells were washed three times with TETBS buffer, and the sera from allergic and healthy donors were added at various dilutions to the TETBS with 20% goat serum (50 μl per well). The plate was incubated on a shaker for 1 hour at room temperature.

After incubation, the plate was washed three times with TETBS buffer, and solutions of monoclonal antibody conjugates with 20% goat serum were added at various concentrations (50 μl per well). The plate was incubated on a shaker for 30 minutes at room temperature. The wells were then washed 6 times with TETBS buffer by alternating between short (10 sec) and long (5 min) washes, as well as 3 times for 10 seconds with TBS buffer. A PCR mixture was introduced into the wells (35 μl per well) and covered with mineral oil (30 μl per well). The composition of the PCR reaction was the following: 35 μl of the amplification reaction mixture containing 3.5 μl of 10х buffer (750 mМ Tris-HCl, pH 8.8, 200 mМ ammonium sulfate, 0.1% Tween-20), 0.25 mМ of each dNTP, 0.6 μМ of primers, 0.3 μМ of fluorescent probe, and 2.5 units of Taq-polymerase (DNA-Technology, Russia).

Quantitative PCR (qPCR) was performed on a DTprime amplifier (DNA-Technology, Russia) using a protocol of initial denaturation for 5 min at 94°C, followed by 40 cycles of the following: 15 sec of annealing and elongation at 60°C and 5 sec of denaturation at 94°C. At each cycle, the fluorescent signal from the probe was measured at 520 nm (FAM channel). Cq values were automatically determined for all reactions using amplifier software (RealTimePCR v.7.9.5.25). The average value of the threshold cycle (Cq) and standard deviation were calculated for each sample.

The detection threshold was calculated as three standard deviations for Cq, which is the value of the threshold cycle in negative samples. The threshold cycle difference (ΔCq) for different samples was calculated using the equation, *ΔCqi* = [*average* (*Cq* − *Cqi*)], where Cqi is a value of the threshold cycle in the sample being studied. Samples with a ΔCq below the detection threshold were considered as negative, and those with a ΔCq greater than or equal to the detection threshold were considered positive. The serum titer was evaluated as the maximum serum dilution at which the corresponding sample was considered positive.

iPCR was performed for the detection of IgM antibodies to Le^C^ as described previously [19]. One-half of the plate wells was coated with the polymer derivative antigen (Le^C^-PAA), and the other half was coated with pure polymer (PAA). After washing, a blocking solution was added (1% BSA, 1% casein, and 1 μM oligonucleotide for blocking in TBS), followed by the addition of samples with a known content of anti-Le^C^ IgM (3 wells with Le^C^-PAA and 3 wells with PAA).

In the case of streptavidin supramolecular complexes, the detection was performed using biotinylated anti-IgM antibodies, followed by interaction with DNA-streptavidin complexes and qPCR analysis. In the case of covalent conjugates, the detection was performed using the conjugates, followed by qPCR. Next, the difference between the Cq values was calculated as follows for each sample added to the wells coated with glycan-free PAA Cq (PAA)(i) (background) and Le^C^-PAA: *ΔCq*(*i*) = *Cq* (*PAA*)(*i*)–*Cq* (*LeC* − *PAA*)(*i*). Samples with ΔCq less than or equal to zero were considered negative, and those with values greater than zero were positive. Only ΔCq values were compared in different experiments.

### Enzyme-linked immunoassay (ELISA)

ELISA of IgE was performed for the recombinant allergen Alt a 1 in the blood serum of an allergic patient as described previously [17].

### Statistical analysis

The average and standard deviation were calculated using Excel software, which was also used to plot the Calibration curves.

## Results

### Optimization of the covalent conjugate preparation technique by the example of anti-IgM antibodies

#### Control of the azido group introduction into antibody molecule (Ab-N3 DOL determination)

The azido group introduction was controlled by reaction with DBCO-sCy5. The initial concentration of the MA2 antibody in all cases was 1 mg/ml, and the reaction was carried out in a carbonate buffer (0.1 M, pH 8.3) for 1 hour at room temperature. The excess DBCO-sCy5 was removed by gel filtration. The degree of labeling of antibody by the azido group (Ab-N3 DOL) was determined as the molar ratio of Cy5 (650 nm) to antibody (280 nm) with the following equation: ***Ab*** − ***N*3** *DOL* = (*ACy*5 * ε*Ab*)/(*AAb* − *ACy*5 * *CF*280) * *εCy*5. A is the absorption at the corresponding wavelength, and CF_280_ is a correction factor (CF_280_ = 0.04). The extinction coefficients for the antibody (ε_Ab_, 280 nm) and sCy5-DBCO (εCy5, 650 nm) were assumed to be 210,000 L * mol^-1^ * cm-1 and 251,000 L * mol^-1^ * cm^-1^, respectively. The results are presented in Table 1.

**Table 1:** The average degree of MA2 antibody labeling with an azido group depending on STP-N3 excess.

Excess of STP-N3, multiplicity	1.5	3	5	8	11	20
Ab-N3 DOL	0.9	1.5	2.3	2.9	3.8	6.4

#### ODN-DBCO reactivity monitoring (ODN-DBCO DOL determination)

As a result of analytical RP-HPLC of the oligonucleotide reaction with dibenzocyclooctyne-activated ester, two peaks of reaction products were obtained with a total yield of 80%. Spectrophotometric analysis of the conjugates of DBCO and sCy5-N3 derivatives demonstrated that each of the products is reactive with a yield of 70%. Thus, the content of reactive ODN-DBCO oligonucleotide was about 60%. Since both reaction products were reactive, we did not perform chromatographic purification of the dibenzocyclooctyne derivative of the oligonucleotide.

ODN-DBCO DOL was defined as the molar ratio of ODN to Cy5 in the ODN-DBCO reaction product with Cy5-N3 at room temperature overnight. Excess Cy5-N3 was removed by double reprecipitation of the reaction product in acetone/lithium perchlorate. The molar ratio of ODN to Cy5 was determined spectrophotometrically. The coefficients of the molar extinction of ODN and Cy5 were 613,500 L * mol^-1^ * cm^-1^ (260 nm) and 250,000 L * mol^-1^ * cm^-1^ (650 nm), respectively, and the correction factor Cy5 for 260 nm was 0.03. Thus, in a conjugation reaction with the azidated antibody, a mixture of ODN and DBCO-NHS reaction products was used after removing the excess reagent by gel filtration and determining the content of the reactive group.

#### Optimization of ODN-DBCO excess and monitoring of conjugation reaction

The optimal excess of ODN-DBCO sufficient for conjugation was determined using an antibody concentration of 1 mg/ml and reaction at room temperature overnight. We investigated the completeness of the conjugation reaction under these conditions with different excesses of ODN-DBCO (1× (equimolar), 2×, 4×, and 8×). To achieve this, an antibody with Ab-N3 DOL = 6.4 was obtained using a 20-fold excess of STP-N3. ODN-DBCO was added at ratios of 1:1, 1:2, 1:4, and 1:8 (by active groups) to equal aliquots of the antibody.

After performing the conjugation reaction with ODN, a 3-fold excess of sCy5-DBCO was added to the reaction mixture. This substance interacts with the azido groups of the modified antibody that were unreacted in the previous step (reaction with ODN-DBCO) for 24 hours at room temperature. The conjugate was isolated on a Superdex 200 10/30 Increase column. The completeness of the reaction (η) of the antibody with ODN was calculated from the molar ratio of ODN and Cy5 in the reaction product using the following equation: *η* = (*A*260 / *εODN*)/ ((*A*260 / *εODN*) + (*A*650 / *εCy*5)) * 100%. The extinction coefficients for ODN (ε_ODN_, 260 nm) and sCy5-DBCO (ε_Cy5_, 650 nm) were taken to be 613,500 L * mol^-1^ * cm^-1^ and 251,000 L * mol^-1^ * cm^-1^, respectively. The results are presented in Table 2.

**Table 2:** Completeness of cycloaddition reaction under given conditions at different ratios of MA2 antibody and ODN by active groups.

Antibody: ODN ratio by active groups	1:1	1:2	1:4	1:8
Completeness of reaction	80%	96%	100%	100%

**Fig 2:**
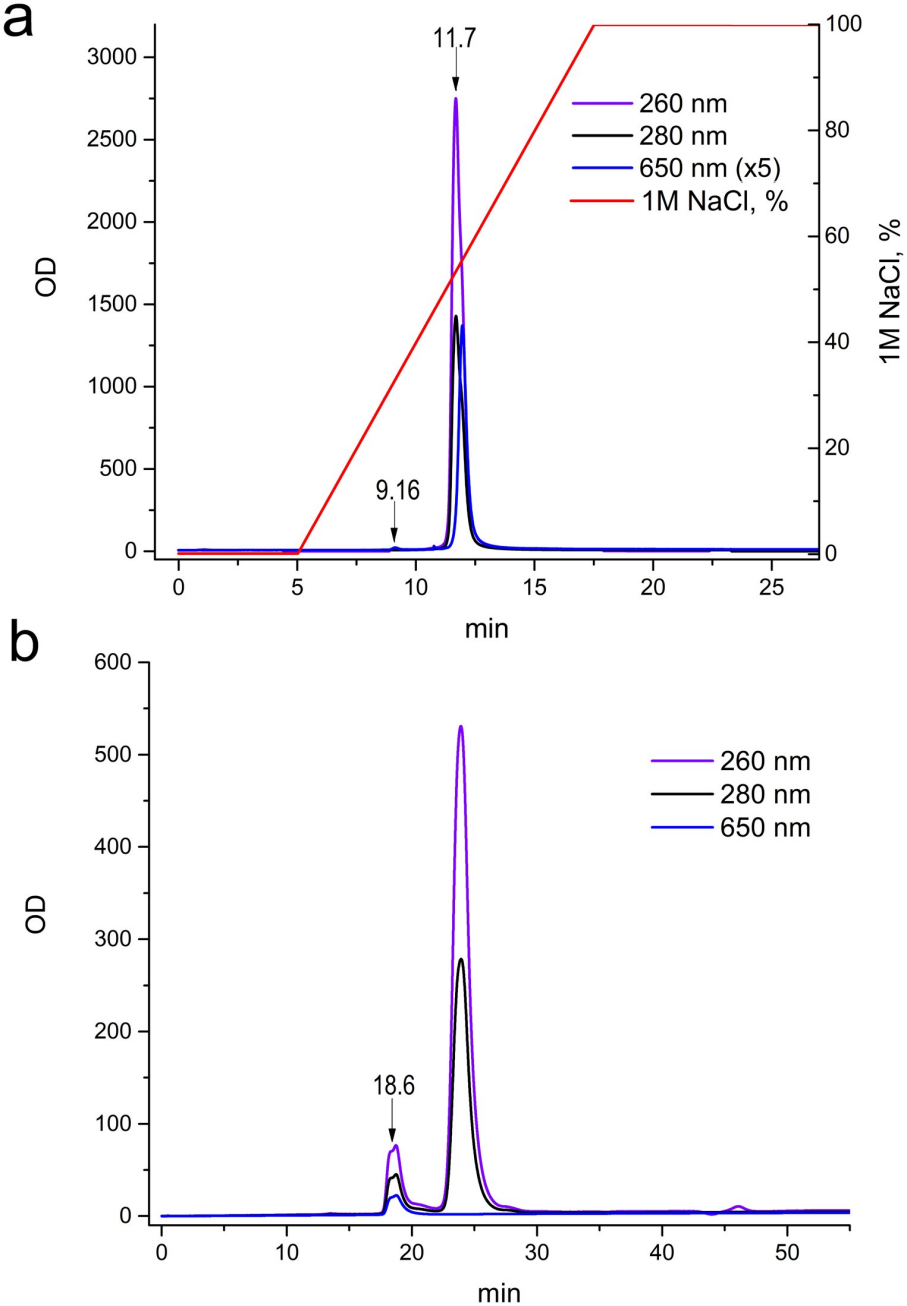
Anion-exchange (a) and gel-filtration (b) chromatograms of antibody (Ab-N3 DOL = 6.4) and oligonucleotide 1:1 reaction products treated with 3-fold excess of sCy5-DBCO. (a) 9.16 min: unmodified antibody; 11.7 min: conjugates and unreacted ODN. (b) 18.6 min: conjugates; 22–27 min: unreacted ODN.

An aliquot of the 1:1 reaction mixture was placed in a RESOURCE Q anion exchange column. There was almost a complete absence of unmodified antibody, as shown in Fig 2a, and the retention time was 9.16 min. The results of the gel-filtration of the 11.7-min peak from the anion exchange column are shown in Fig 2b, and the retention time of conjugate is 18–20 min. Later, a 4-fold excess of ODN was used to ensure the completeness of the reaction under given conditions.

#### Preparation and testing of conjugates with different antibody-ODN ratios

**Fig 3:**
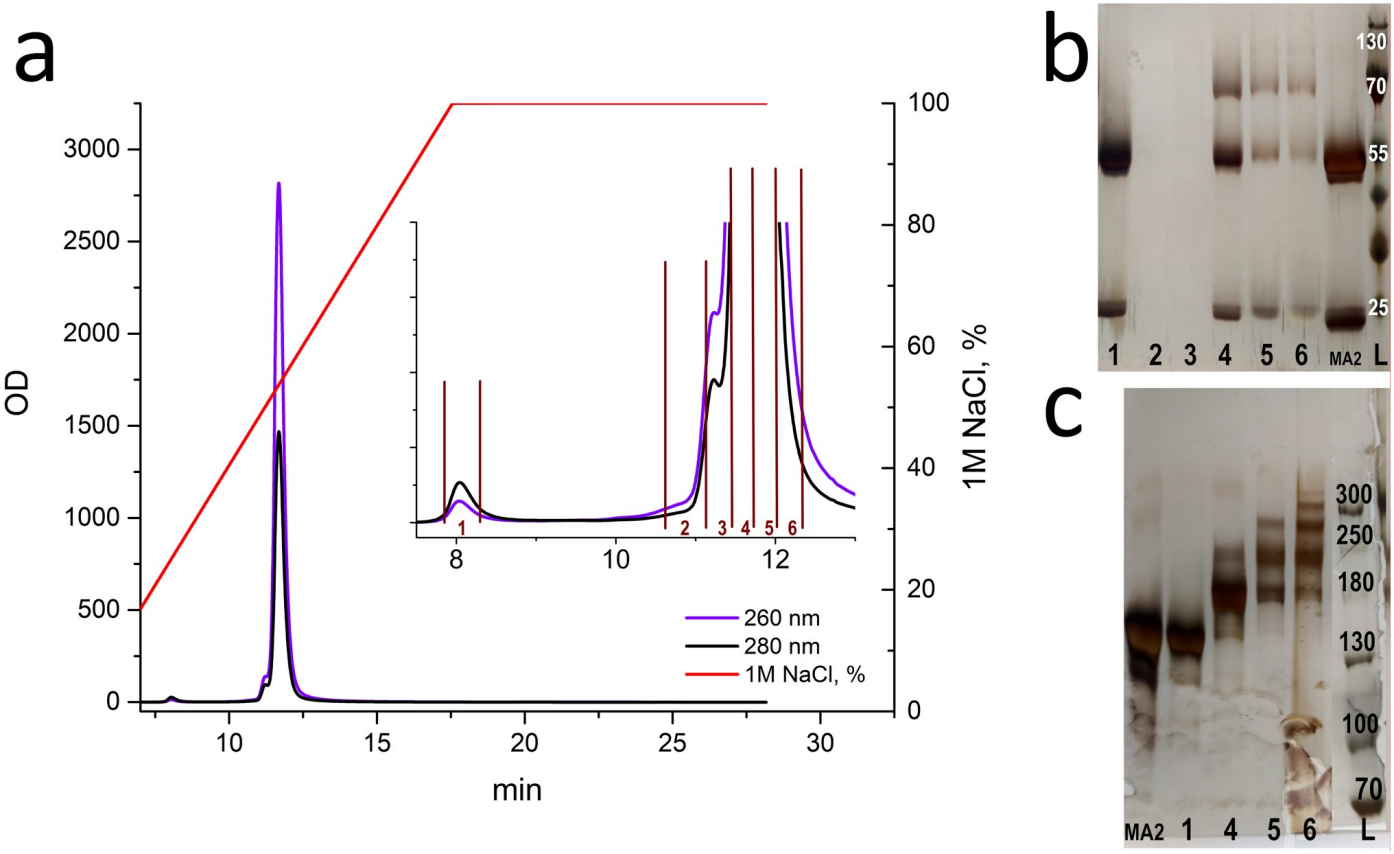
Results of anion-exchange chromatography obtained by purification of conjugation reaction products involving an antibody with Ab-N3 DOL = 1.1. (a) Anion-exchange chromatogram of conjugation reaction products involving an antibody with Ab-N3 DOL = 1.1. The numbers of fractions are indicated on the enlarged fragment (fraction 1: unreacted antibody; fractions 2–6: peak 11–12.5 min). Right: SDS-PAGE in reducing (b) and non-reducing (c) conditions. The sample numbers correspond to the fractions, MA2 is the original antibody. L: protein ladder (b: PageRuler Prestained Protein Ladder, 10 to 180 kDa; c: Spectra Multicolor Broad Range Protein Ladder).

Conjugates were obtained with different degrees of antibody labeling with oligonucleotide (mono-, bi-, tri-, etc.) in the antibody azidation by using 2- and 6-fold excesses of STP-N3. Ab-N3 DOL for these antibodies was 1.1 and 2.6, respectively. These results are in good agreement with the data in Table 1. The conjugation was carried out by mixing and incubating the solutions of the azidated antibody and oligonucleotide with DBCO overnight at room temperature. After determining the amount of active reaction groups (Ab-N3 DOL and ODN-DBCO DOL), the excess of oligonucleotide introduced into the reaction was calculated. In each case had a 4-fold excess of reactive ODN-DBCO relative to the azido groups of the antibody. The presence of unreacted azide groups of the antibody was tested by introducing a 10-fold excess of sCy5-DBCO. Fig 3 presents a chromatogram from the RESOURCE Q anion exchange column and the electrophoregram obtained during the first stage of purification of the reaction product of azidated MA2 antibody (DOL 1.1) with ODN-DBCO.

The chromatogram demonstrates the presence of unreacted antibody (peak 1). Fractions 2 and 3 showed no bands of free or labeled antibodies on the electrophoregram (the concentration is below the sensitivity of the detection method). Fraction 4 contains a monolabeled antibody, and fraction 5 contains mono- and double-labeled antibodies. Fraction 6 mainly contains double- and triple-labeled antibodies, but it also contains mono-, quadruple-, and even quintuple-labeled antibodies.

The conjugation reaction was done using 250 μg of azidated antibody. The antibody from peak 1 did not react with sCy5-DBCO, suggesting that there were no azido groups in the antibody structure. The amount of antibody without azido groups was 60 μg, which corresponds to 25% of the amount of antibody taken in the conjugation reaction. Thus, with a labeling degree of antibody Ab-N3 DOL equal to 1.1, up to 25% of it remains unchanged. This indicates a wide distribution of the labeling degree and suggests the presence of up to 25% of the antibodies with two ODNs in the reaction mixture. Therefore, we were not able to obtain a monolabeled antibody with a high yield under these conditions.

**Fig 4:**
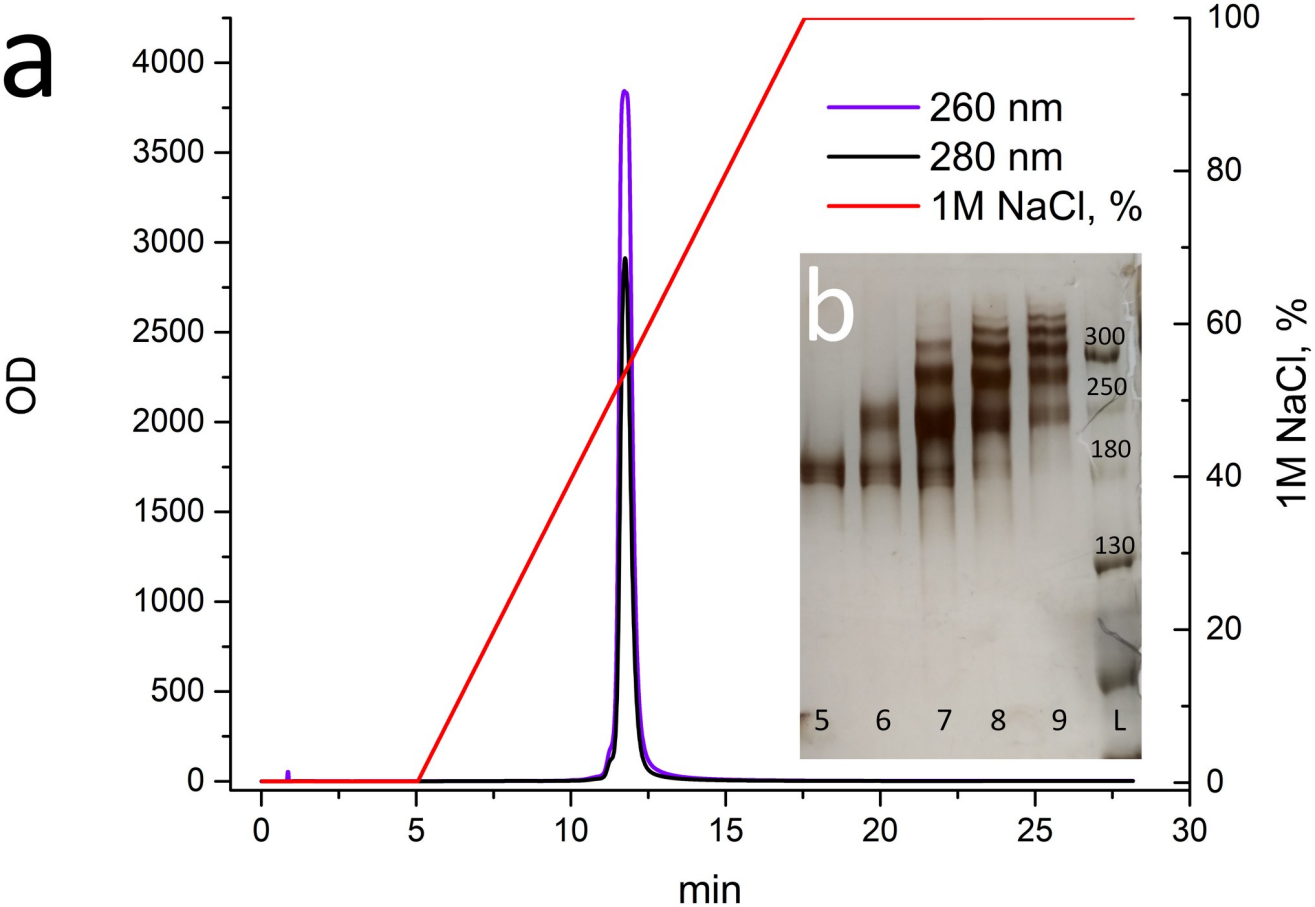
Results of anion-exchange chromatography obtained during purification of the conjugation reaction products involving an antibody with Ab-N3 DOL = 2.6. (a) Anion-exchange chromatography of the conjugation reaction products involving an antibody with Ab-N3 DOL = 2.6. (b) SDS-PAGE under non-reducing conditions. 5–9: fractions collected between 11 and 13 minutes. L: Spectra Multicolor Broad Range Protein Ladder.

To increase the yield of the conjugate, it would be reasonable either to obtain a mixture of mono- and double-labeled antibodies (Ab-N3 DOL nearly 1.5) or to reuse (modify) the antibody from fraction 1. After a chromatographic purification (Fig 4a) on the RESOURCE Q anion exchange column, fractions were isolated from the reaction mixture with the antibody **MA2** Ab-N3 **DOL = 2.6**. The distribution of the number of labels per antibody varied from 1 (fraction 5, about 180 kDa by the length marker) to 6 (fractions 8 and 9) (Fig 4b). There was no peak corresponding to the unreacted antibody.

**Fig 5:**
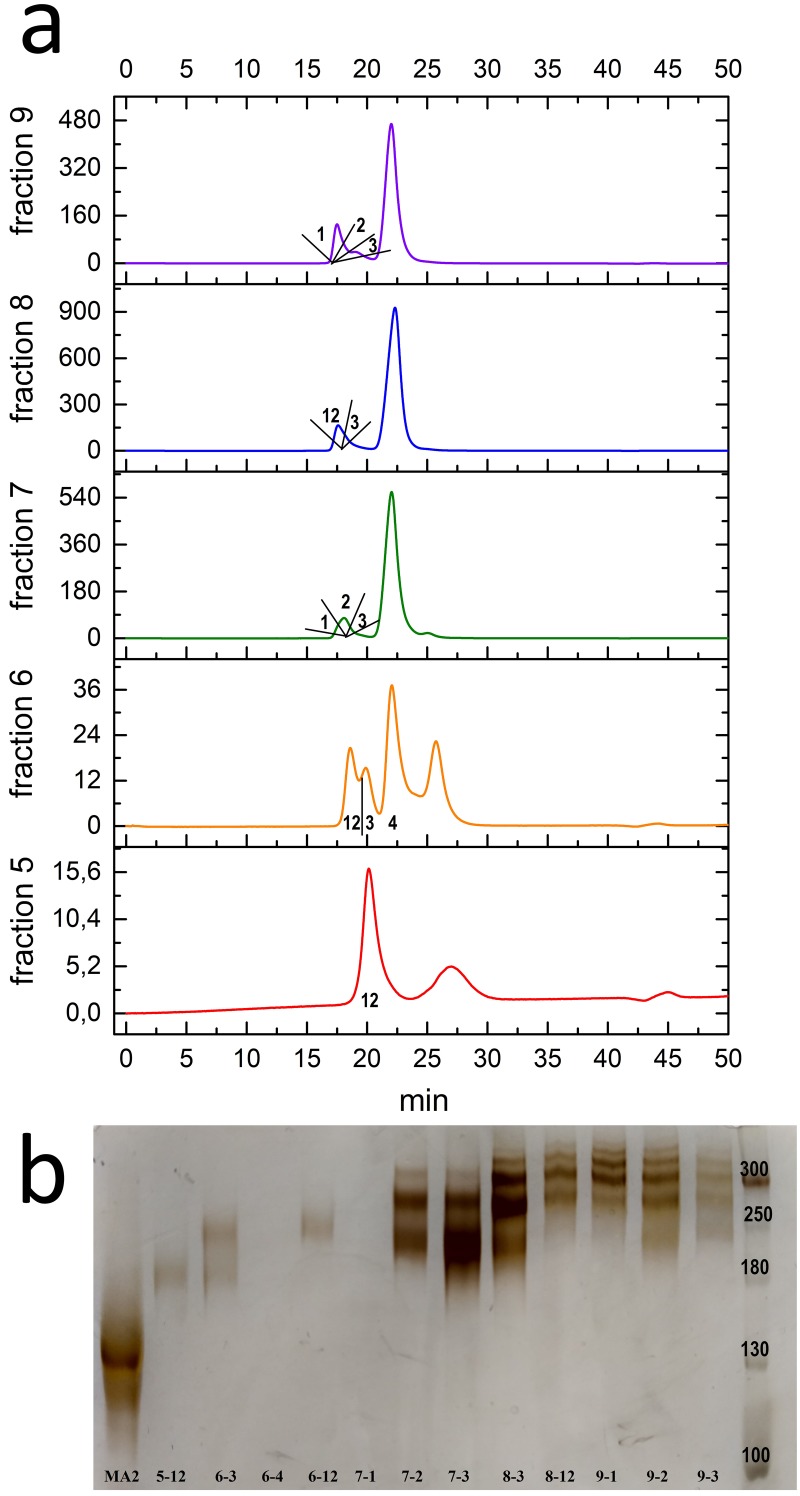
Results of the second stage of purification of conjugation reaction involving antibody Ab-N3 DOL = 2.6. (a) Gel-filtration chromatography of fractions obtained after anion-exchange chromatography (Fig 4) of conjugation reaction with the antibody Ab-N3 DOL = 2.6. (b) Electrophoregram of fractions collected during gel-filtration chromatography. The track numbers correspond to the designated fractions. MA2 is the original antibody. L: Spectra Multicolor Broad Range Protein Ladder.

Subsequently, all fractions were purified by gel filtration on a Superdex 200 10/30 Increase column to remove the unbound oligonucleotides. The electrophoregram of the fractions obtained is shown in Fig 5. One can see that the repeated fractionation of the target peak allows for the isolation of fractions with an exact label content. Among the obtained fractions, we have chosen the following fractions for use in iPCR: fraction 5–12, which contains one label per antibody molecule according to the results of electrophoresis; fraction 7–3, which contains two labels per antibody molecule (with an admixture of 3 labels); fraction 8–3, which contains three labels per antibody molecule (with an admixture of 2 and 4 labels); and fraction 9–1, which contains four to five labels per antibody molecule.

#### Quantitative determination of IgM antibodies to the Le^C^ disaccharide in serum using the iPCR method with covalent MA2-ODN conjugate

**Fig 6:**
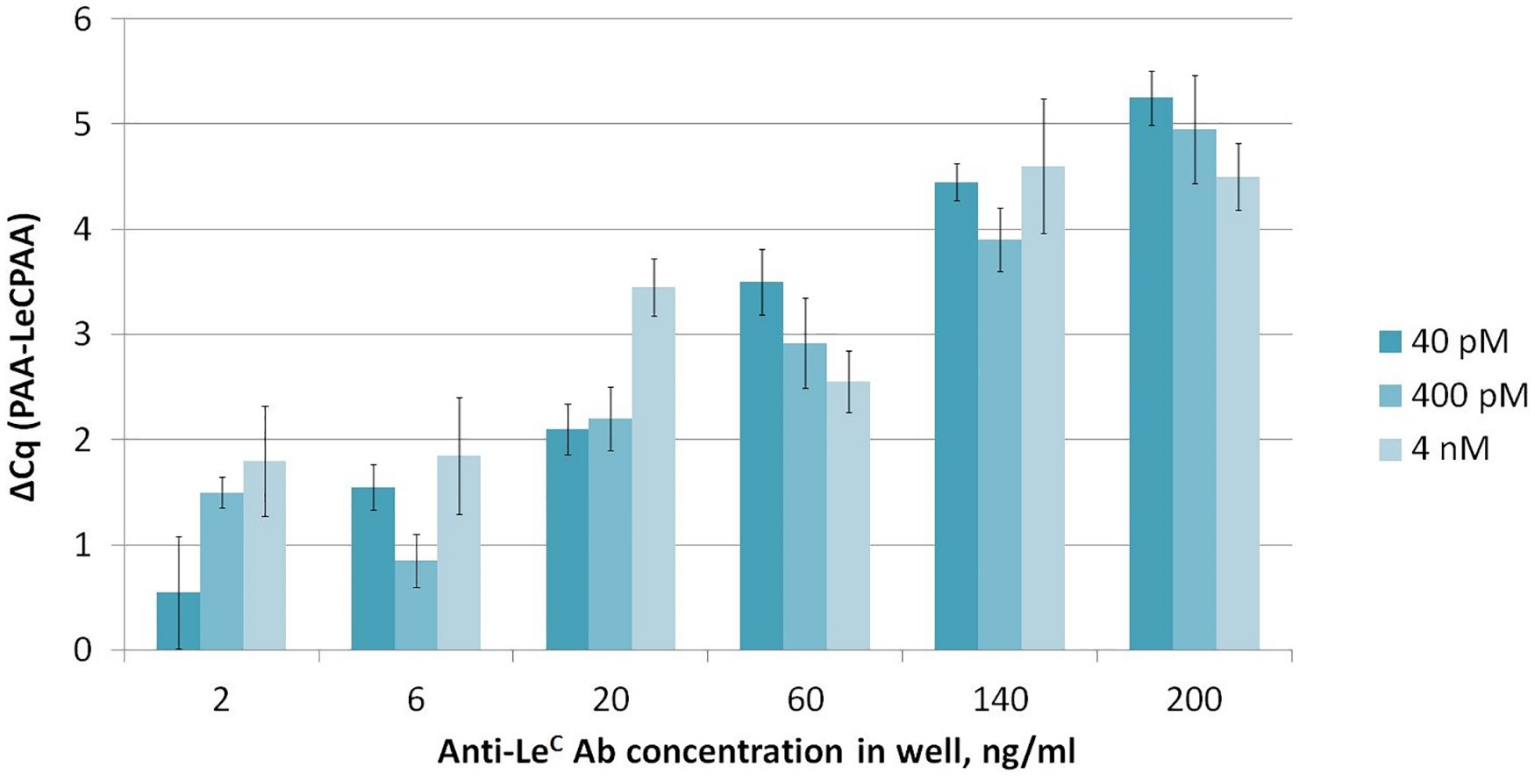
Results of monolabeled conjugate testing (concentrations: 40 pM, 400 pM, and 4 nM) by iPCR of samples with a known concentration of anti-Le^C^ IgM.

To select an optimal working dilution of conjugates for iPCR, qPCR data were first obtained for 100-fold titrations of conjugates in deionized water. qPCR was performed with dilutions of conjugates and ODN solutions with known concentrations (100 pM and 1 pM) as standards (qPCR was performed in three repeats with standards and in two repeats with conjugates dilutions). The efficiency of the PCR reaction was 92%. The concentration of the initial solutions of conjugates varied from 0.7 to 4.4 μM. Dilutions of the conjugate with one label (fraction 5–12; Fig 5) with final concentrations of 40 pM, 400 pM, and 4 nM were used for iPCR. The iPCR was performed according to the procedure described in Materials and methods (Fig 6).

The highest value of ΔCq (5.25) for an anti-Le^C^ concentration of 200 ng/ml was obtained using a conjugate concentration of 40 pM. For concentrations of 400 pM and 4 nM, values of 4.95 and 4.5 were obtained, respectively. The background value Cq(PAA) directly depends on the conjugate concentration used in iPCR. Thus, the average Cq(PAA) was 19.4 for 4 nM, 21.5 for 400 pM, and 25.3 for 40 pM. Therefore, we set the working conjugate concentration as 40 pM, which provides a low background signal and the largest ΔCq for a sample with a high concentration of anti-Le^C^ IgM.

### Testing of conjugates with different numbers of oligonucleotides per antibody

#### Conjugates for the detection of anti-Le^C^ IgM antibodies in serum

The following conjugates were selected: 5–12 with one label per antibody; 7–3 with two labels (with an admixture of 3 labels); 8–3 with three labels (with an admixture of 2 and 4 labels), and 9–1 with four to five labels. In the first stage, an attempt was made to align the conjugates by antibody content, for which the titrations of the conjugates were tested in qPCR. The quantity of oligonucleotide was converted to the quantity of antibodies based on an evaluation of the ratio presented in electrophoregrams. The following concentrations of conjugates by ODN were used in iPCR (the antibody concentration is the same): 1 label: 40 pM; 2 labels: 80 pM; 3 labels: 120 pM; 4–5 labels: 180 pM.

**Fig 7:**
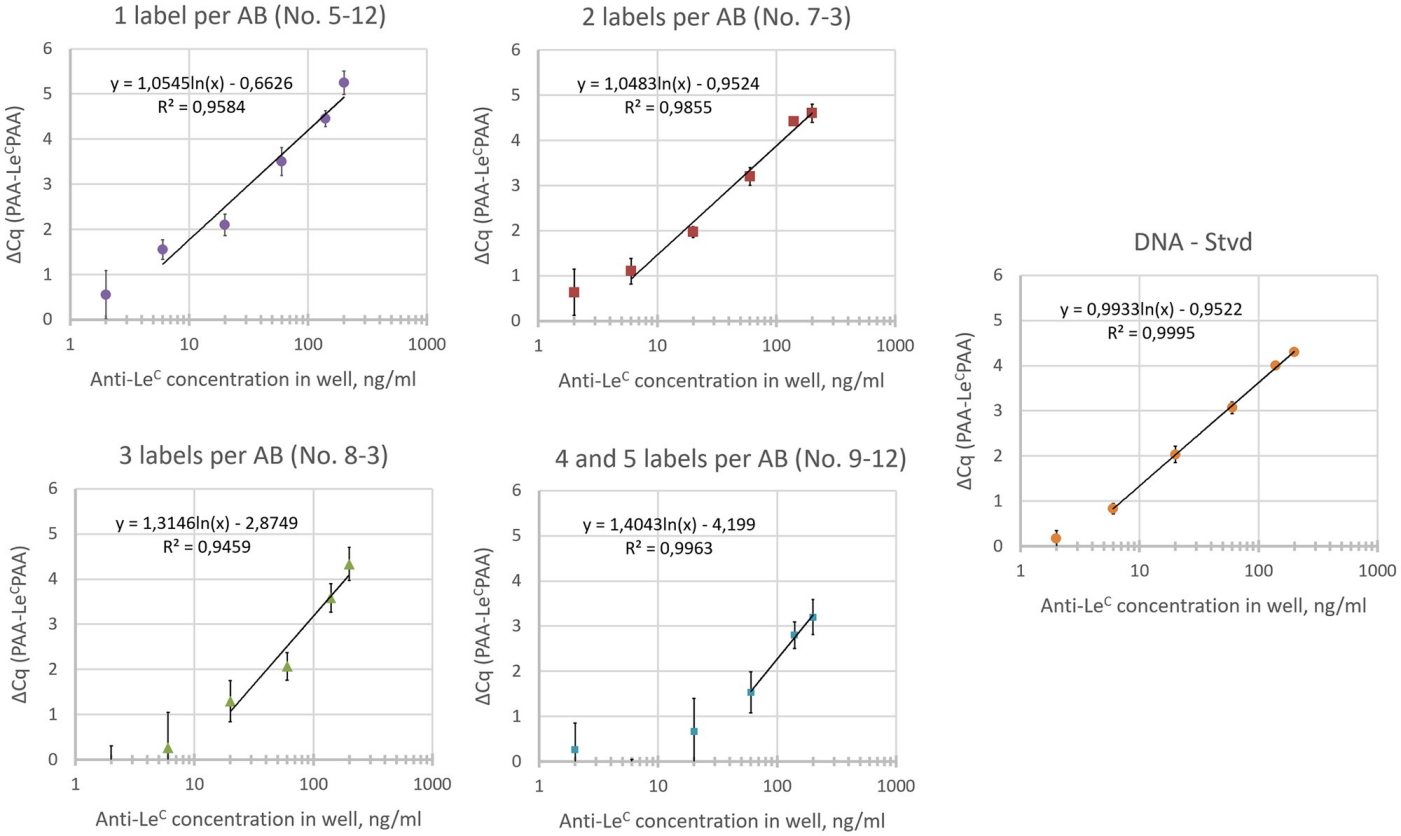
Results of the different degrees of labeling conjugates tested by iPCR on a panel of samples with a known concentration of anti-Le^C^ IgM. The numbers of labels per AB corresponds to conjugates with the same number of ODN molecules per antibody. DNA-Stvd is a variant of iPCR using a streptavidin supramolecular complex [19].

Taking into account the data obtained from the electrophoregram, we can consider the concentration of the antibody and antibody conjugate as a whole to be almost the same for all conjugates (approximately 40 pM). The average background value of Cq (PAA) for all conjugates during iPCR was 22.7–24.6. Fig 7 shows the results of testing the conjugates in the iPCR on a panel of samples with a known concentration of anti-Le^C^ IgM, as well as a comparison with the DNA-streptavidin complex (DNA-Stvd) used earlier.

Conjugates with 1 and 2 labels exhibited the same sensitivity as the DNA-streptavidin conjugate (6 ng/ml). For a 3-label conjugate, the lowest detectable target concentration was 20 ng/ml, and that for a conjugate with 4 and 5 labels was 60 ng/ml. Accordingly, the sensitivity of the method decreases drastically with the increase of the labeling degree of the detecting antibody in this case. There was no noticeable difference for conjugates with 1 and 2 labels. Bearing in mind the wide distribution of antibody labeling with the azido group, we believe that obtaining an antibody with an Ab-N3 DOL of 2 is optimal. This will ensure a 100% conversion of the antibody to the conjugate with the main product being double-labeled.

#### Conjugate for the detection of IgE antibodies in serum

**Fig 8:**
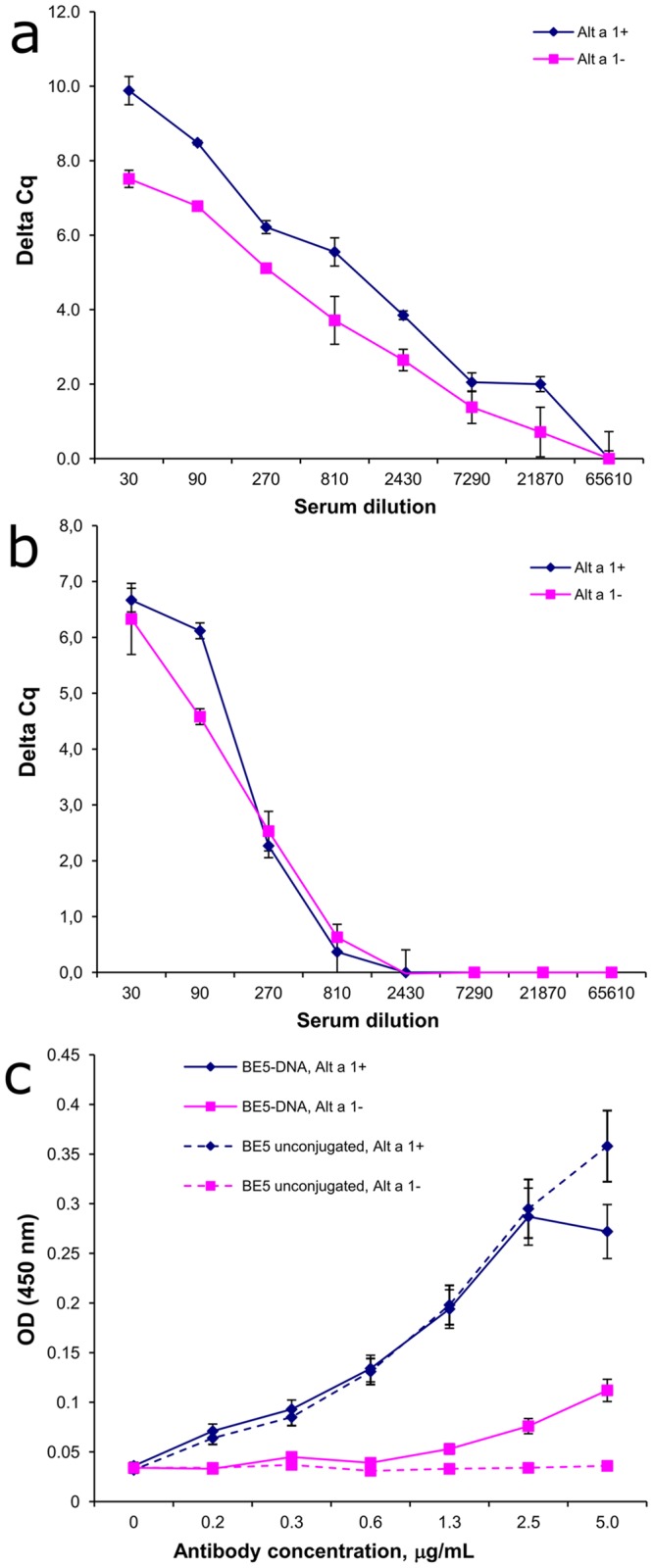
Titration curves of allergic and healthy donor sera obtained from the iPCR analysis of IgE on the recombinant allergen Alt a 1. (a) Titration curves of allergic sera. (b) Titration curves of healthy donor. Data are shown for the BE5-ODN conjugate with two labels at a concentration of 10 ng/ml concentration. (c) Titration curves of the BE5-ODN conjugate and unconjugated antibody BE5 in ELISA when analyzing the serum of an allergic patient (20-fold dilution). The assay was performed on strips with and without sorbed antigen.

Conjugates of ODN—BE5 antibody to human IgE were obtained as described above for the conjugate of anti-IgM antibodies, so a detailed description of the results is not provided. A conjugate with two labels was used in iPCR. In the case of the BE5-ODN conjugate, we found a non-specific binding to blood serum components of both allergic and healthy donors (Fig 8). This phenomenon was observed in iPCR analysis in both the presence and absence of the sorbed Alt a 1 allergen at concentrations of the BE5-ODN conjugate of tens of ng/ml (Fig 8a and 8b). When analyzing the allergic patient serum by ELISA, a similar nonspecific signal was also observed in the 20-fold dilution, but at higher concentrations of ODN-BE5 conjugate, the signal was on the scale of micrograms per milliliter (Fig 8c). We did not observe this phenomenon in the case of unconjugated antibody (Fig 8c) and BE5-streptavidin-DNA complex [18], as well as in the case of incubation with unconjugated oligonucleotide. Therefore, we believe that the observed nonspecific binding is a result of changes in the structure of the antibody molecule during its chemical modification.

To eliminate nonspecific binding, we tried to optimize the conditions of the immunoassay and introduced an additional blocking step between the stages of the sorption of allergen and the addition of the analyzed serum (compared to the method described for the DNA-streptavidin complex [18]). Blocking was performed with TBS buffer with various components: 1% BSA, 10% goat serum, 0.73 mM sucrose, 0.5% tween-20, 0.02% tween-20 with 1% BSA, 2% skim milk, 0.5% triton X100, 0.1% gelatin, and 1% human immunoglobulin. However, the introduction of an additional blocking step did not allow us to eliminate nonspecific binding.

## Discussion

### Attainment, isolation, purification, and analysis of conjugates

Covalent DNA-antibody conjugates were obtained using the cycloaddition reaction of azides and alkynes. This necessitated the introduction of an azide group into the antibody. We chose to introduce the azido group into the antibody at the amino group via 4-sulfotetrafluorophenyl (STP) ether, which reacts with amino groups like NHS ethers and forms a strong amide bond [21]. This method is one of the simplest ones available.

Previously, we attempted to obtain a conjugate by the cycloaddition reaction of azides and alkynes catalyzed by monovalent copper (the "copper click" method). However, the complex of monovalent copper with THPTA often leads to precipitation when mixed with the antibody solution (although not for all antibodies). This is consistent with the fact that copper ions can cause protein denaturation [8]. When selecting the reaction conditions for human IgG preparation, we succeeded in identifying conditions under which the antibody did not precipitate at a low catalyst concentration. However, with a 20-nucleotide ODN model with a linear alkyne, only 30% of the antibodies were labeled. In contrast, when using the 60-nucleotide ODN with a linear alkyne, no conjugate was obtained at all if the reaction mixture was kept in argon atmosphere for the entire conjugation time.

The main problem was the isolation of the conjugate and its purification from unreacted ODN. We did not succeed in isolating the conjugate by anion-exchange chromatography on the RESOURCE Q anion exchange column. Although the retention time of unreacted azidated antibody is much less than that of ODN (9.16 and 11.7 min, respectively), the conjugate is not eluted between them as expected. Furthermore, at 11 min, mono- and double-labeled conjugates are eluted before the main peak of the ODN, and 4-5-labeled conjugates are eluted after the main peak of the free ODN (there was no peak separation at 260 nm). The chromatography was performed in a wide range of pH (4.5–7.5), but this did not improve the separation of the conjugate and ODN in a linear gradient of NaCl.

We performed a reaction with pentynoic acid sulfotetrafluorophenyl ester at pH 8.3. At this pH, both the N-terminal α-amino group (pKa 8.95) and the ε-amino group of lysine (pKa 10.53) were reactive and are referred to as reactive amino groups [22, 23]. Analysis of the resulting conjugates indicated that when the azido group is introduced into the antibody using STP or NHS esters, there was a wide distribution of the labeling degree of antibody by the azido group. The average degree of antibody labeling by the azido group only statistically reflects the presence of labels on the antibody. Thus, it was shown that when Ab-N3 DOL is 1.1, 25% of the antibodies contained no azido groups, up to 25% contained 2 azido groups, and up to 50% contained one azido group (Fig 3). The selected scheme for the purification of the reaction products allows for the removal of the unmodified antibody at the anion-exchange chromatography stage and the excess ODN at the gel filtration stage. Nevertheless, it was impossible to obtain only the mono-labeled conjugate with a yield exceeding 50%. Obtaining a predominantly double-labeled conjugate in this case seems to be more rational.

Gong et al. obtained similar labeling degrees of antibody with a similar excess of DBCO-PEG5-NHS reagent [9]. The use of four molar equivalents of DBCO-PEG5-NHS resulted in 2.3 DBCO molecules per antibody after the reaction. In turn, this yielded a conjugate with 1.2 ODNs per antibody after reaction with a 4-fold excess of azidated oligonucleotide (45 bases). Unfortunately, the authors did not explain the incomplete involvement of DBCO-groups in the conjugation. They investigated the effect of the unlabeled antibody in the conjugate and showed that the 12.5% impurity of unlabeled antibody in the conjugate does not affect Cq.

It should be noted that a sharp decrease of ΔCq (a decrease of the target signal) was observed for only the content of impurity of the unlabeled antibody above 400% relative to the conjugate content. In the range of 25–400%, the decrease of ΔCq does not exceed one. We did not study the tolerance of our systems of the presence of unlabeled antibodies, despite the possibility of not performing the purification from unreacted antibody in the case of such a strong tolerance detection. Van Buggenum determined the position of the reactive lysine residues in mouse IgG upon modification by a 5-fold excess of tetrazine-NHS [10]. It turned out that there residues in the heavy chain were reactive (12 lysine residues in the molecule comprising 2 in the light chain and 10 in the heavy one). Unsurprisingly, all the reactive lysine residues were on the antibody surface and were available to the solvent.

Thus, the usage of NHS (or STP) chemistry did not allow us to produce an antibody labeled with a strictly defined number of labels or at least with a small spread in the number of labels—we always obtained a mixture. To solve this problem, it seems necessary to introduce the azido group in a different way. It is likely that methods based on the modification of glycan residues would improve the situation. Monoclonal antibodies contain one conserved N-glycosylation site at position N297 in each heavy chain. In this case, about 20% of the antibodies also contain a second N-glycosylation site in the variable region of the heavy chain [24]. Glycan motifs on antibodies are diverse (up to 30 variants), and monoclonal antibodies in culture can be synthesized in various glycoforms [25]. Nevertheless, enzyme processing can produce a homogeneous glycan composition [26]. The best solution for recombinant antibodies would be to use modified aminoacyl-tRNAs that carry amino acids with introduced functional groups that are inserted into a particular site during translation [27]. Another alternative is the SpyTag/SpyCather technique [28].

### iPCR with covalent conjugates

#### Anti-IgM-ODN conjugate for anti-Le^C^ antibody detection in sera

The usage of a supramolecular complex and covalent direct conjugates on the example of the anti-Le^C^ IgM detection system resulted in similar sensitivity for mono- and double-labeled conjugates and the supramolecular complex (Fig 7). It should be noted that the protocol based on direct conjugates is shorter by several stages (1 hour of incubation and 3 wash steps). However, the same result can be achieved by using pre-prepared and gel-filtered DNA-biotin-streptavidin complexes with introduced antibodies, which were first described by Niemeyer in 1999 [29]. The increase of the labeling level of antibody in the example of the anti-Le^C^ IgM detection system led to a decrease of the sensitivity, which was first observed at 3 ODN molecules per antibody and showed no significant difference between the conjugates with one and two labels.

#### Anti-IgE-ODN conjugate for anti-allergen antibody detection in sera

iPCR assay of anti-IgE conjugates showed that the specificity of the BE5 antibody changed. We used DBCO-PEG_4_-NHS for ODN modification, which is similar to what Gong et al. used for antibody modification [9]. They did not observed any changes in specificity of the antibodies. We expected that a higher solubility of the reagent and the extended linker between dibenzocyclooctyne and NHS-group would reduce the effect of ODN on the affinity and specificity of the antibodies. However, the specificity of the BE5-ODN conjugate prepared using the DBCO-PEG_4_-NHS reagent also changed.

The positions of the reactive amino groups where azido groups are introduced followed by the attachment of ODN seem to be very important. The position of the lysines available for the reaction of amino groups on the surface of the antibody is specific for each antibody. This was confirmed by the fact that we obtained conjugates with the MA2 antibody with preserved antibody specificity if the antibody amino groups were modified, and so did other authors with several other antibodies. It is should be noted that the biotinylated BE5 antibody did not change in specificity after streptavidin was added to the supramolecular complex of 5'- and 3'-biotinylated ODN and streptavidin [18]. The biotinylation of antibody BE5 was carried out with the N-hydroxysuccinimide ester of biotin attached to the same reactive amino groups as STP-N3. Nevertheless, further streptavidin binding in the ODN-complex did not affect the specificity of the BE5 antibody.

## Conclusions

We obtained covalent DNA-antibody conjugates using SPAAC reaction, and the method for the preparation and purification of the conjugates was optimized. The possibility of using these conjugates in iPCR was analyzed. After modification of the antibodies by amino groups, it was impossible to obtain monolabeled antibodies or antibodies with a strictly defined number of labels. The degree of labeling determined by the dyes introduced to azido groups only statistically reflected the final result.

One of the two monoclonal antibodies used in this study (anti-IgE BE5) lost its specificity after covalent (direct) conjugation with ODN. The sensitivity for the detection of IgM antibodies against Le^C^ by iPCR using covalent conjugate proved to be similar to that measured for iPCR using the supramolecular complex of 5'- and 3'-biotinylated ODN and streptavidin, which we examined in a previous study [19]. However, the procedure became two steps shorter and at least 1 hour faster.

## Supporting information

S1 Fig SDS-PAGE in non-reducing (a) and reducing (b) conditions for fractions collected during anion-exchange chromatography purification of the conjugation reaction products involving an antibody with Ab-N3 DOL = 2.6. (TIF)
