# Supplementary figures and images for: Development of the covalent antibody-DNA conjugates technology for detection of IgE and IgM antibodies by immuno-PCR
Source: PLoS One. 2019 Jan 4;14(1):e0209860. doi: 10.1371/journal.pone.0209860 (PMC6319726; doi:10.1371/journal.pone.0209860)

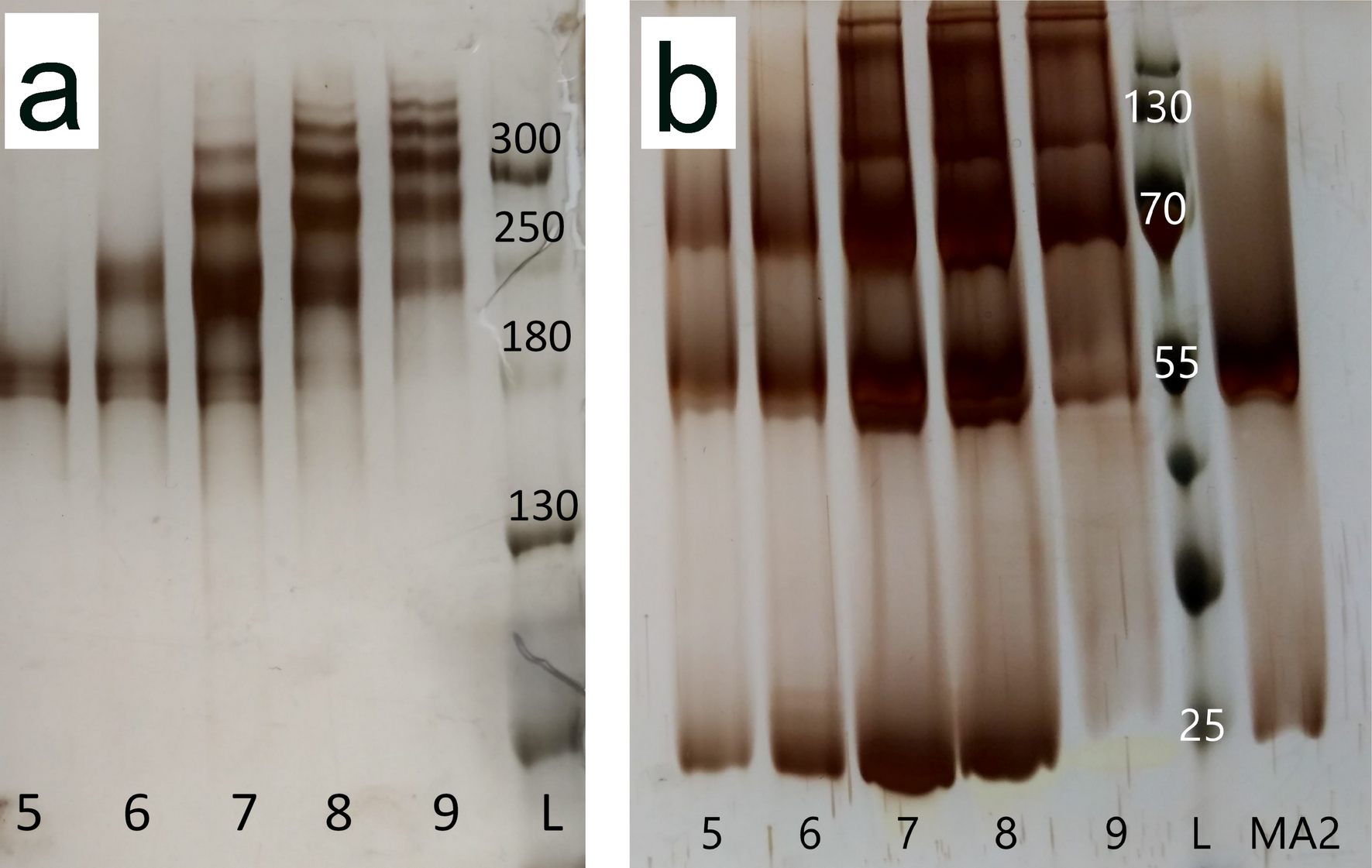

Supplement: S1 Fig — (TIF) [file pone.0209860.s001.tif]
